# Effectiveness of Nature-Based Mindfulness Interventions to Improve Mental Health: A Narrative Review

**DOI:** 10.3390/ijerph23050551

**Published:** 2026-04-24

**Authors:** Costanza Vecchio, Chiara Copat, Paola Rapisarda, Gea Oliveri Conti, Margherita Ferrante

**Affiliations:** 1 Department of Medicine, Surgery and Advanced Technologies “G.F. Ingrassia”, Via S. Sofia 87, 95123 Catania, Italy; ccopat@unict.it (C.C.); paolarapisarda5@gmail.com (P.R.); olivericonti@unict.it (G.O.C.); margherita.ferrante@unict.it (M.F.); 2 School of Specialization in Public Health and Preventive Medicine, University of Catania, Via S. Sofia 87, 95123 Catania, Italy

**Keywords:** nature-based mindfulness interventions, mental health, one health, ecological awareness, climate risk, resilience

## Abstract

**Highlights:**

**Public health relevance—How does this work relate to a public health issue?**

- Nature-based mindfulness interventions (NBMIs) reduce stress, anxiety, depressive symptoms and rumination in vulnerable groups.
- NBMIs strengthen core resilience mechanisms, relevant in the face of increasing environmental and climate related stressors.

**Public health significance—Why is this work of significance to public health?**

- Interventions conducted in forests, gardens and other biodiverse environments enhance the effects of conventional mindfulness-based programmes by activating restorative biophysical pathways and fostering nature connectedness.
- Integrating mindfulness with sustainability-oriented curricula links contemplative practices with systems thinking, environmental ethics and pro environmental behaviours, offering an innovative approach for One Health-aligned interventions.

**Public health implications—What are the key implications or messages for practitioners, policy makers and/or researchers in public health?**

- Scaling up low cost, culturally adaptable NBMI programmes could strengthen public mental health strategies, particularly in Mediterranean and climate vulnerable regions.
- NBMIs offer a dual benefit—supporting mental wellbeing while promoting ecological awareness—making them a promising component of policies that aim to enhance resilience in both humans and ecosystems.

**Abstract:**

**Background:** Human health is closely interconnected to our ecosystem. Several studies found evidence that nature-based interventions improve mental health. Very recently, these approaches have started including mindfulness practices. Nature-based mindfulness interventions (NBMIs) combine contemplative practices with exposure to natural environments and are increasingly recognised as promising tools for supporting mental health and resilience within a One Health perspective, fostering physio-psychological wellbeing whilst promoting nature awareness and a sense of connection with our planet—“biofilia”, as defined by American biologist Edward Wilson. Given the growing psychological impacts of climate-related stressors, NBMIs may offer particular value for regions with high climate-risk and ecological vulnerability. **Methods:** A narrative literature review was conducted following established principles for high-quality non-systematic reviews. A non-systematic but structured search of PubMed, Scopus, Web of Science and Cochrane Library (January 2018–November 2025), complemented by grey literature, identified studies involving adolescents and adults participating in interventions integrating mindfulness practices with natural environments. Extracted data included study context, participant characteristics, intervention type, mental health and resilience outcomes. **Results:** Across heterogeneous designs, NBMIs consistently reduced stress, anxiety, depressive symptoms and rumination, while improving sleep, vitality, attention and self-regulation. Most studies reported enhanced nature connectedness—an important mediator of wellbeing and pro-environmental behaviour. Programmes delivered to disaster-affected populations showed reductions in distress. **Conclusions:** NBMIs are feasible, low-cost and adaptable interventions with dual benefits for mental health and ecological awareness. They offer promising One Health-aligned strategies for strengthening psychological resilience in climate-vulnerable regions, warranting further research and context-specific adaptation.

## 1. Introduction

Within a One Health framework, human wellbeing is understood as inseparable from the health of ecosystems. The One Health framework is an integrated, unifying approach that recognises the interdependence between human health, animal health, and ecosystem integrity. It emphasises that interactions among humans, domestic and wild animals, plants, and the broader environment shape patterns of health, vulnerability, and resilience, particularly in the context of climate change and environmental degradation [1,2]. The One Health approach promotes holistic responses to complex health challenges, contributing to both population wellbeing and the protection of ecosystems [2]. Environmental degradation—through biodiversity loss, pollution, land-use change, or climate-related hazards—alters patterns of disease, increases exposure to physical and psychosocial stressors, and erodes the ecological conditions that support population health [2,3]. Conversely, intact ecosystems contribute to physical, mental, and social wellbeing, acting as buffers against stressors and providing spaces for restoration, social connection, cultural identity, and long-term resilience [4,5]. Protecting ecosystem integrity is therefore not only an ecological imperative but a key public health strategy, particularly in regions such as the Mediterranean area that are highly vulnerable to climatic instability and environmental decline.

Mental health is a fundamental component of human health and sustainable development. Yet despite its substantial societal and economic burden, mental health remains under-prioritized and under-resourced in many health systems worldwide. In the context of converging global crises—including climate change, economic uncertainty, social fragmentation, and forced migration—strengthening mental health governance, financing, and accessible community-oriented services has become increasingly urgent [6]. Mental health arises from complex interactions among biological, psychological, social, and environmental determinants. Environmental stressors—heat waves, extreme weather events, landscape degradation, chronic noise, air pollution, and urban crowding—are now well-recognized contributors to psychological distress, trauma, and loss of wellbeing [7,8]. At the same time, accumulating evidence demonstrates that contact with natural environments provides measurable psychological benefits, supporting stress regulation, cognitive restoration, immune function, and emotional wellbeing through multiple biophysiological pathways [9,10].

The “biophilia hypothesis,” articulated by Edward O. Wilson (1992) [11], proposes that humans possess an innate predisposition to attend to and affiliate with the natural world—a tendency rooted in evolutionary processes and increasingly supported by empirical research [12]. Biodiverse, undisturbed ecosystems appear to amplify psychological and physiological benefits, reinforcing the notion that environmental health directly shapes human health. Immersive exposure to forests, coastal areas, and other natural settings has been shown to improve cardiovascular function, reduce stress via modulation of the hypothalamic-pituitary-adrenal axis, enhance immune activity, and support metabolic regulation, partly through interactions with volatile organic compounds (phytoncides), environmental microbiota, negative air ions, sensory stimuli, and opportunities for physical activity and mindful attention [10,13].

Parallel to this scientific development, **mindfulness-based interventions (MBIs)** have gained recognition as evidence-based strategies to reduce stress, anxiety, and depressive symptoms [14]. *Mindfulness* is commonly defined as the capacity to bring intentional, non-judgmental awareness to present-moment experience [15,16]. Although MBIs are delivered in secular formats, they derive conceptually and methodologically from long-standing contemplative traditions, most notably Buddhist psychology. Contemplative traditions encompass a broad family of meditation and mental training approaches aimed at fostering ethical awareness, emotional balance and deeper understanding of human experiences through the cultivation of sustained attention, present-moment awareness and compassion [17,18]. Over recent decades, some contemplative practices have been adapted into secular and evidence-based formats, most notably through mindfulness-based interventions, and integrated into clinical contexts, education, and public health settings [15,16,19]. Since the development of Mindfulness-Based Stress Reduction (MBSR) by Jon Kabat-Zinn in the late 1970s, representing a paradigm shift through the integration of contemplative practices into Western biomedical and behavioral science, and the subsequent introduction of Mindfulness-Based Cognitive Therapy (MBCT) in the 1990s by Zindel Segal, Mark Williams, and John Teasdale, which advanced the clinical application of mindfulness, MBIs have been increasingly implemented across healthcare, educational, and community settings. Their pioneering work has generated a robust and growing empirical evidence base, contributed to the standardization and manualization of mindfulness-based approaches, and informed clinical guidelines and public mental health policy. As a result, MBIs are now widely adopted in contexts such as the UK National Health Service, schools, workplaces, prisons, and youth services [20].

Emerging research suggests that when practiced in natural environments, mindfulness may yield enhanced health benefits, whilst fostering a sense of connection with nature and ecological awareness. Studies in environmental psychology show that higher levels of mindfulness are associated with stronger nature connectedness and more sustainable attitudes and behaviours, likely through enhanced present-moment awareness and greater relational and ethical sensitivity [12,21,22]. Mindfulness practices, particularly when embedded in natural environments, may further foster feelings of interdependence, belonging, and care for the natural world, while simultaneously strengthening individual wellbeing [4,23]. Recent sustainability-oriented frameworks explicitly position mindfulness as a catalyst for ecological awareness and inner–outer transformations, linking individual wellbeing, environmental stewardship, and collective resilience within a One Health perspective [24]. **Nature-Based Mindfulness Interventions** (NBMIs) combine contemplative practices with direct sensory immersion in natural settings, potentially increasing attentional regulation, emotional balance, physiological restoration, and connectedness to nature. These interventions may also strengthen ecological awareness and pro-environmental behaviours, supporting the idea that psychological wellbeing and the human–environment relationship are mutually reinforcing [23]. As individuals cultivate a sense of belonging within broader ecological systems, motivation to protect and care for natural environments may increase -an outcome that aligns closely with One Health principles and with the environmental and climate challenges affecting regions at high climate-risk.

In this context, **the aim of this narrative review** was to explore how NBMIs are currently implemented across countries and settings, and how they may contribute to mental health, resilience, and ecological awareness within a One Health perspective. Specifically, we sought to:

(1) describe the types of nature-based mindfulness interventions applied to promote mental health in adults and young adults/adolescents;
(2) examine their use in contexts relevant to environmental and climate-related vulnerability, including Mediterranean and comparable ecosystems; and
(3) identify key mechanisms and opportunities at the interface of mental health, ecological connectedness, resilience, and One Health.

## 2. Materials and Methods

### 2.1. Study Design and Methodology

This article is based on a **narrative literature review** adopting a structured and integrative approach aimed at conceptually mapping and critically interpreting heterogeneous evidence in an emerging and methodologically diverse field. Rather than aiming for an exhaustive, protocol-driven systematic review, we adopted an exploratory and integrative approach, considered appropriate for a field that is still emerging, heterogeneous in terminology, and methodologically diverse. It was conducted in accordance with recommendations aimed at improving transparency and methodological quality of narrative reviews, particularly those outlined in the Scale for the Assessment of Narrative Review Articles (SANRA) [25].

Explicit attention was given to clearly defining the review aims, search strategy, eligibility criteria, and interpretative procedures.

### 2.2. Search Strategy and Information Sources

A structured but non-systematic literature search was conducted between May and November 2025, encompassing scientific publications published from January 2018 to November 2025. The selected timeframe was chosen to capture recent developments linking mindfulness, environmental exposure, mental health, and climate related resilience within contemporary One Health perspectives. Electronic databases consulted included PubMed, Scopus, Web of Science, and Cochrane Library. The search was complemented by targeted searches in grey literature (e.g., institutional reports, NGO documents, and conference proceedings) where appropriate. Grey literature was primarily consulted to inform background sections and to contextualise an emerging and still fragmented field, particularly where peer-reviewed empirical evidence remains limited. Consequently, grey literature did not contribute to study inclusion decisions and was not subjected to formal quality appraisal procedures. All findings presented in Section 3 are therefore based exclusively on peer-reviewed empirical studies. This explicit description of information sources and search procedures is provided to enhance transparency in line with key quality considerations commonly applied to narrative reviews, as outlined by SANRA [25].

Search terms combined three main conceptual domains:

- **Nature/environment** (e.g., nature-based, green space, forest, wilderness, natural environment, outdoor, ecotherapy);
- **Mindfulness/contemplative practices** (e.g., mindfulness, mindfulness-based, meditation, mindful walking, MBSR, MBCT);
- **Mental health/psychological outcomes** (e.g., mental health, stress, anxiety, depression, wellbeing, resilience).

These terms were adapted to the syntax of each database and combined using Boolean operators (e.g., “mindfulness” AND “nature-based” AND “mental health”). The search process was iterative, allowing emerging concepts identified during screening to inform subsequent searches.

### 2.3. Eligibility Criteria

Because this is a narrative review, inclusion criteria were intentionally broad, with the goal of capturing the diversity of interventions and settings. We considered sources that met the following criteria:

- **Population:** Adults and adolescents (≥10 years), including both clinical and non-clinical populations.
- **Intervention:** Any intervention combining exposure to natural environments (e.g., parks, forests, gardens, coastal or rural areas) with mindfulness-based or contemplative practices (e.g., formal meditation, mindful walking, mindful gardening, structured mindfulness programmes).
- **Outcomes:** Mental health–related outcomes (e.g., stress, anxiety, depression, psychological distress, wellbeing, resilience, quality of life) and, when available, physiological (e.g., heart rate variability, cortisol) or interpersonal/functional outcomes (e.g., work functioning, social connectedness).
- **Setting/context:** Studies conducted in any country were considered.
- **Type of evidence:** Primary empirical studies (quantitative, qualitative, or mixed-methods), as well as conceptual and theoretical papers that explicitly addressed nature-based mindfulness interventions in relation to mental health, resilience, or One Health.
- **Language and timeframe:** Publications in English available between 2018 and November 2025.

We excluded:

- Interventions that involved nature exposure without any mindfulness-related component.
- Interventions using virtual natural environments/virtual reality.
- Studies where mindfulness was delivered without a meaningful nature-based or environmental component.
- Studies not including mental health outcomes.
- Articles that only mentioned nature or mindfulness in passing without describing an intervention or mental-health-related outcomes.
- Commentaries or editorials with no substantive description or analysis of interventions.

### 2.4. Study Selection and Data Extraction

Although PRISMA guidelines were not formally applied due to the narrative design, transparent selection procedures were adopted to enhance methodological clarity. Titles and abstracts were screened to identify potentially relevant studies, followed by full-text assessment against eligibility criteria. Three reviewers independently performed the screening of titles/abstracts and full-text articles. The results were subsequently compared and discussed within the research team to ensure conceptual consistency. Any discrepancies were resolved through consensus discussion. Given the exploratory and narrative nature of the review, all studies meeting the predefined eligibility criteria were retained, and no eligible studies were excluded after full-text assessment.

For each included study or source, we extracted and summarised the following information, where available:

- Country and setting (e.g., urban/rural, coastal, forest, Mediterranean-like environment);
- Characteristics of the population (clinical vs. non-clinical, specific vulnerabilities, e.g., chronic illness, exposure to environmental or climate stressors);
- Description of the nature-based component (type of natural environment, intensity and duration of exposure);
- Description of the mindfulness component (formal vs. informal practices, use of structured programmes such as MBSR/MBCT, individual vs. group format);
- Study design and main mental health outcomes;
- Any reported links to resilience, vulnerability, environmental or climate-related risks, or One Health frameworks.

Data extraction was conducted descriptively to support both accurate representation of individual studies and identification of cross-cutting themes.

### 2.5. Approach to Quality Appraisal

In line with the objectives of a narrative review, a formal exclusion-based risk-of-bias assessment was not applied. Instead, an interpretative quality appraisal was conducted to evaluate methodological transparency and conceptual coherence across studies.

Particular attention was given to:

- Clarity in the description of the intervention (both mindfulness and nature-based components);
- Transparency of methods (e.g., sampling, outcome measures, follow-up);
- Appropriateness of the mental health outcomes to the stated aims;
- Coherence between the theoretical framework (e.g., biophilia, One Health, resilience) and the intervention design.

This judgement informed the weight and interpretative emphasis given to each study in the narrative synthesis, rather than being used as a basis for exclusion.

### 2.6. Narrative Synthesis

Because of the heterogeneity in study designs, populations, types of natural environments, and outcome measures, Evidence was synthesised using a thematic and concept-driven narrative approach, organised around:

- Types of nature-based mindfulness interventions (e.g., brief single-session practices, structured weekly programmes, residential interventions);
- Populations and vulnerabilities addressed (e.g., individuals with stress, anxiety, depression, substance use, chronic physical illness, or exposure to environmental/climate stressors);
- Mental health and physiological outcomes, including indicators of resilience and wellbeing;
- Links to One Health and ecological awareness, such as interventions explicitly aiming to enhance connection with nature, biophilia, or eco-consciousness.

The synthesis aimed to identify models of implementation, core components, promising practices, and gaps in the current evidence regarding nature-based mindfulness interventions, with a focus on how these interventions are applied across different countries and settings and how they may contribute to mental health, resilience, and ecological awareness within a One Health perspective.

## 3. Results

### 3.1. Literature Search Results and Overview of Included Studies

In the study selection, a total of 398 records were identified from the following databases: PubMed (*n* = 281), Cochrane Library (*n* = 8), Scopus (*n* = 64), and Web of Science (*n* = 45). After removing 78 duplicate records, 320 records were screened for eligibility. Of these, 26 were excluded because they were review articles. Among the remaining 294 studies, 12 were selected based on the inclusion criteria.

The included studies were published between 2018 and November 2025 and were conducted across a range of geographical contexts, including Europe (United Kingdom, Denmark, Sweden, the Netherlands), East Asia (South Korea, China), and North America (United States), reflecting growing international interest in nature-based mindfulness interventions (NBMIs). Overall, the studies involved approximately 1200 participants, with sample sizes ranging from 6 to 276. Participants spanned a wide age range from early adolescence (12 years) to older adulthood, and included university students, community samples, vulnerable populations (e.g., individuals with mental health conditions or cognitive impairment), disaster-affected individuals, and clinical populations. Interventions were delivered across diverse settings, such as therapeutic gardens, forests, urban parks, temples, residential retreat centres, academic environments, healthcare facilities, and community spaces.

Study designs were heterogeneous and comprised randomised controlled trials, non-randomised experimental studies, longitudinal observational studies, qualitative investigations, mixed-methods approaches, and pilot feasibility studies. The studies are presented according to level of evidence, to facilitate interpretation (Table 1). Detailed study designs, interventions characteristics, methodological information, and expanded results are described in Appendix A (Table A1). Randomised controlled trials (*n* = 4) compared NBMIs with indoor, urban, or active control conditions, primarily among university students, and evaluated outcomes including stress, rumination, mood, sleep quality, mindfulness, wellbeing, and nature connectedness [26,27,28,29]. Quasi-experimental and pre–post studies (*n* = 4) examined more heterogeneous and vulnerable populations, including adolescents, older adults, individuals with mental health conditions, and disaster-affected communities. Interventions ranged from brief single-session practices to short intensive forest-based programmes [30,31,32,33]. Observational and non-randomised experimental studies (*n* = 2) investigated psychophysiological and attentional mechanisms under controlled conditions [34,35], while qualitative studies (*n* = 2) provided in-depth insights into self-regulation, emotional balance, and resilience processes [36,37].

Together, the included studies form a heterogeneous but coherent evidence base supporting the relevance of NBMIs for mental health and ecological awareness within a One Health framework.

### 3.2. Mental Health Outcomes: Depression, Anxiety, Stress

Across the reviewed literature, reductions in depression, anxiety, and perceived stress emerged as the most consistently reported outcomes of nature-based mindfulness interventions (NBMIs). Improvements were observed in community samples, university students, clinically vulnerable individuals, and populations exposed to environmental stressors, such as natural disasters [27,28,30,31].

Comparative studies showed that identical mindfulness curricula delivered outdoors were more likely to produce sustained stress reductions and lower rumination than indoor or built-environment equivalents; a cross-study pattern indicates that while mindfulness-based interventions delivered in both natural and non-natural settings can alleviate distress, exposure to natural environments may enhance the magnitude, efficiency, or durability of stress reduction, particularly under conditions of chronic or cumulative stress [26,27].

Importantly, brief and intensive formats—including two-day forest therapy programmes and five-day residential retreats—generated medium to large reductions in distress, comparable to effects typically reported for longer programmes such as standard eight-week MBSR courses [27,31]. This suggests that immersive exposure to natural settings may accelerate therapeutic effects, particularly for individuals experiencing acute stress. However, several studies noted attenuation of benefits after intervention cessation, highlighting the importance of continued practice and to maintain regular access to restorative environments for long-term impact [28].

### 3.3. Mindfulness, Self-Regulation and Attention and Restorative Benefits

Beyond symptom outcomes, the synthesis highlights mindfulness-related processes as central mechanisms of change in NBMIs. In multiple studies, increases in mindfulness functioned as mediators or moderators linking nature-based engagement to improvements in wellbeing, life satisfaction, and stress recovery [30,31].

Evidence suggests that natural environments may support mindfulness through attentional restoration and reduced cognitive effort, particularly for individuals experiencing high mental load. Studies comparing indoor focused-attention training with outdoor open-monitoring approaches demonstrated that nature-supported mindfulness better preserved attentional gains and reduced executive fatigue over time, especially among stressed participants [34].

Residential retreats and brief interventions alike were associated with enhanced self-regulation, decentring, and emotional balance, with some effects persisting beyond the intervention period [27,32]. Although not all studies observed setting-related differences in mindfulness outcomes, the evidence indicates that exposure to natural settings potentially strengthening its stability, integration, and regulatory effects.

### 3.4. Qualitative Insights on Emotional Wellbeing and Resilience

Qualitative work further clarified how nature-based mindfulness fosters self-regulation and resilience across different contexts.

In this review, resilience is conceptualised not simply as an absence of distress but as a dynamic capacity for adaptive self-regulation in the face of psychosocial and environmental stressors, which both complements and extends traditional mental health indicators such as depression, anxiety, and stress. From this perspective, resilience is closely linked to mindfulness-related processes, such as awareness, decentring, and emotional flexibility, that enable individuals to respond more adaptively to life challenges. Across qualitative evaluations, participants described a progression, from relief from everyday demands, to cultivation of mindfulness attitudes (e.g., non-judgement, acceptance, kindness), to deeper emotional insight and self-regulation [36,37].

Nature was consistently considered as an active co-regulator, supporting calm, presence, and emotional safety through multisensory engagement, silence, and slow movement. Importantly, participants linked these experiences not only to immediate wellbeing but to enhanced coping in daily life, describing increased capacity to manage stressors, maintain emotional balance, and adapt flexibly to ongoing demands, months after intervention completion [37].

Together, these findings suggest that NBMIs strengthen internal capacities, including awareness and emotional regulation, that underpin resilience and adaptive responses to psychosocial and environmental adversity, a key consideration particularly in contexts of environmental and psychosocial adversity.

### 3.5. Connectedness to Nature

A consistent pattern across interventions was the enhancement of connectedness to nature, with implications for both mental health and pro-environmental behaviour.

Controlled trials showed that only nature-based conditions reliably increased nature connectedness over time, alongside reductions in rumination and stress [26,27]. Qualitative accounts reinforced this, suggesting that strengthened connection fostered a sense of belonging, comfort, meaning, and emotional grounding [36,37].

Experimental evidence further indicates that environmental characteristics matter: water-based landscapes, especially when paired with reflective or creative practices, elicited stronger physiological relaxation and deeper cognitive and emotional engagement than forest or rock settings [35]. These findings point toward the relevance of environmental qualities in optimising restorative outcomes, with implications for urban planning and public health policies.

### 3.6. Sustainability-Oriented Mindfulness

#### 3.6.1. Eco-Wellness Programmes

A subset of interventions explicitly extended mindfulness beyond individual mental health to address sustainability-related behaviours and planetary health.

Pilot studies of mindfulness-based eco-wellness programmes integrating contemplative practice with education on food systems, energy use, transportation, and consumption demonstrated high feasibility and acceptability across community and clinical settings [33].

These programmes showed preliminary improvements in mental health outcomes, such as anxiety, depression, fatigue, alongside modest shifts toward pro-environmental behaviours, such as dietary changes and modest reductions in carbon related activities. Although controlled trials are lacking, these findings suggest the potential for co-benefits across human and environmental health, a central goal of the One Health paradigm.

#### 3.6.2. Mindfulness–Sustainability Programmes

Conceptual and qualitative work on mindfulness programmes explicitly oriented to sustainability highlighted that integrating mindfulness with systems thinking, ethics and nature connected practices may help shift mindsets, deepen engagement and support inner–outer transformations needed for societal responses to climate and ecological crises [38]. While empirical outcome data are still limited, these approaches signal emerging directions for the field. Wamsler et al. (2026) [38] examined how mindfulness-based curricula can be aligned with contemporary sustainability and polycrisis challenges. Their qualitative analysis focused on two pioneering training programmes for mindfulness teachers: the Mindfulness-Based Sustainable Transformation (ST) course, an eight-module online teacher-training programme combining mindfulness and compassion practices with the Inner Development Goals, systems thinking, behavioural science and Indigenous knowledge; and the MBSR & EcoAwareness (EA) workshop series, a six-session online course designed to help certified MBSR teachers integrate eco-metaphors, nature-based practices, contextual framing of stress within planetary crisis, and ethical–justice perspectives into standard MBSR teaching. The findings underscore that mindfulness–sustainability integration emphasises embodiment, relational awareness and ethics, positioning mindfulness not only as a tool for individual stress regulation but also as a resource for cultivating resilience and meaningful engagement amid escalating environmental crises. Although Wamsler et al. (2026) [38] does not report clinical or quantitative mental-health outcomes and is therefore not included in Table 1, it is incorporated into the narrative synthesis due to its strong conceptual relevance to the aims of this review. The study provides an empirically informed framework for integrating mindfulness with sustainability, ecological awareness and inner–outer transformation. Its inclusion helps situate outcome-based NBMIs within a broader One Health-oriented transformation context, without conflating conceptual and clinical evidence.

### 3.7. Implications for Vulnerability, Resilience and One Health

Taken together, these findings suggest that nature-based mindfulness and meditation programmes can reduce distress (depression, anxiety, stress), enhance mindfulness and self-regulation, strengthen feelings of connection with nature and others, and in some cases promote more sustainable behaviours. Their relevance appears particularly pronounced for populations exposed to chronic stress, environmental disruption, or climate-related adversity (e.g., disaster-affected communities, highly stressed students, people with chronic stress-related illness). From a One Health perspective, NBMIs represent integrative, scalable approaches that act simultaneously on mental health and the human–environment relationship, key dimensions particularly in regions facing climate vulnerability, ecosystem degradation, and rising mental health burdens. However, the heterogeneity of intervention formats, outcome measures, and contexts highlights the need for more comparative designs and longer-term evaluations.

## 4. Discussion

The findings of this narrative review suggest that nature-based mindfulness interventions (NBMIs) offer a promising, multidimensional pathway for promoting mental health, strengthening psychological resilience, and advancing ecological awareness within a One Health framework. Consistent with the biophilia hypothesis—which posits an innate human inclination to affiliate with the natural world—NBMIs appear to activate synergistic psychophysiological processes that contribute to wellbeing. Across the included studies, integrating contemplative practice with exposure to natural environments consistently produced meaningful benefits in vulnerable groups, including older adults, students under academic stress, disaster-affected populations, and individuals with chronic or recurrent mental health difficulties. These results highlight the interdependence of human and environmental health, especially in regions such as the Mediterranean area, where climate-related hazards, biodiversity loss, and socioeconomic pressures converge to heighten population-level vulnerability.

A central insight emerging from this review is that nature contact and mindfulness—each independently associated with psychological benefits—may exert amplified effects when combined. Reductions in stress, anxiety, depressive symptoms, and rumination across diverse interventions indicate that NBMIs support attentional regulation, emotional calibration, and cognitive reframing. Sensory engagement with biodiverse environments involves additional biophysical mechanisms—such as exposure to phytoncides, negative air ions, diverse environmental microbiota, and restorative multisensory stimuli that may contribute to autonomic regulation and immune enhancement, consistently with evidence from other nature-based interventions, such as forest therapy [9,10]. This convergence illustrates a core One Health principle: psychological wellbeing is inseparable from the vitality of surrounding ecosystems.

NBMIs also show particular relevance for resilience building in the context of environmental and climate-related stressors. Evidence from populations affected by wildfires, floods, and other disruptions indicates that even brief forest-based or outdoor mindfulness practices can reduce distress and enhance vitality and perceived coping [31]. During periods of disruption—such as the COVID-19 pandemic—mindful outdoor walking supported students manage stress, restore a sense of routine, and reconnect with their surroundings [28]. Qualitative accounts deepen this picture, describing nature as a “co-regulator” that provides sensory stability, emotional containment, and a sense of belonging within a larger ecological whole [36,37]. These relational and embodied processes align with known pathways of resilience development, including increased self-efficacy, emotional flexibility, cognitive reappraisal, and strengthened social and ecological connectedness and may help buffer emerging climate-related psychological burdens such as eco-anxiety, anticipatory grief, and trauma.

Another salient theme concerns nature connectedness, a construct consistently associated with greater wellbeing and pro-environmental behaviour. Interventions that actively engage individuals with natural settings—through mindful movement, gardening, poetry, sensory immersion, or open-monitoring practices—enhanced feelings of awe, belonging, and interconnectedness [4,12,35,36,37]. These experiences are not only beneficial for mental health but also lay a psychological foundation for environmental stewardship. While the included studies were conducted across diverse geographical contexts, the mechanisms identified, such as stress reduction and enhanced nature connectedness, may be relevant for regions facing combined environmental and psychosocial stressors, including areas affected by climate instability, such as Mediterranean regions, where environmental deterioration and heat-related stress are intensifying.

The review also points to a notable evolution towards a one health perspective within the mindfulness field. Recent sustainability-oriented programmes, such as those described by Wamsler et al. (2026) [38], show that mindfulness curricula are increasingly incorporating ecological literacy, systems thinking, and social-environmental ethics. This reflects a broader recognition that psychological resilience is inseparable from ecological resilience, justice, and collective wellbeing. When mindfulness practices acknowledge their contemplative heritage—rooted in traditions emphasising interdependence, compassion, and reverence for life—they naturally align with One Health principles and offer fertile ground for fostering sustainable worldviews.

Despite the encouraging findings, important limitations remain. Many included studies relied on small samples, limited follow-up, or lacked control conditions, underscoring the need for more rigorous research designs. Empirical evidence specific to Mediterranean settings remains limited, despite the region’s distinct climate-related challenges, including heat extremes, drought, water scarcity, and wildfire exposure, highlighting the need for future research to examine the applicability and effectiveness of nature-based mindfulness interventions in this context. Evidence concerning socioeconomically marginalised groups—who often face the highest levels of environmental risk—also remains sparse. Future research should prioritise these populations and incorporate physiological measures (e.g., cortisol, HRV, inflammatory markers) to clarify biobehavioural pathways and the mechanisms underpinning NBMI effectiveness.

## 5. Conclusions

The evidence indicates that NBMIs provide a feasible, low-cost, and culturally adaptable approach to strengthening mental health and resilience amid accelerating climate and environmental change. By simultaneously supporting psychological wellbeing, enhancing nature connectedness, and fostering pro-environmental behaviour, NBMIs offer a unique entry point for integrated One Health interventions. This is especially relevant for climate-vulnerable Mediterranean regions, where intertwined environmental, social, and health challenges call for holistic strategies capable of supporting both human and ecological resilience.

Furthermore, the emerging integration of mindfulness with sustainability science, systems thinking, and contemplative ethics indicates that future interventions may extend beyond symptom reduction to support broader forms of inner–outer transformation essential in the context of the global polycrisis.

NBMIs may represent a promising approach for climate-vulnerable regions, including Mediterranean contexts; however, future studies conducted in these settings are necessary to confirm their applicability and effectiveness under region-specific environmental and social conditions. Public health systems seeking holistic, preventative, and community-based strategies may consider the integration of such interventions to help address the growing mental health burdens associated with climate-related stressors. By reinforcing the interdependence of human and ecosystem health, these interventions embody the core principles of One Health, offering a practical and scalable means to cultivate psychological resilience while nurturing the ecological foundations upon which health ultimately depends.

## Figures and Tables

**Table 1:** Characteristics and main findings of included Nature-Based Mindfulness Intervention (NBMI) studies, organized by level of evidence. Detailed study designs, intervention content, measurement tools, statistical analyses and results are reported in Appendix A (Table A1).

Study	Population	Intervention	Outcomes-Efficacy
**Randomised Controlled Trials (RCT)**			
Choe et al., 2020 (UK) [26]	University students and staff (*n* = 99)	6-week Mindfulness Based Stress Reduction (MBSR) programme (weekly 1 h sessions), delivered in natural outdoor, built outdoor or indoor setting; identical curriculum across conditions	↓ Depression ↓ Anxiety ↓↓ Stress ↓↓ Rumination ↑↑ Mindfulness ↑↑ Nature connectedness (>in Natural setting)
Djernis et al., 2021 (Denmark) [27]	Stressed university students (*n* = 60)	5-day residential MBSR-based retreat conducted outdoors in a therapeutic garden vs. indoors vs. wait-list	↓ Stress ↑ Mindfulness ↑ Self-compassion (= in both interventions) ↑↑ Nature connectedness (in nature retreat)
Ma et al., 2023 (UK) [28]	University students with sleep difficulties (*n* = 104)	7-day mindful walking intervention, nature vs. urban environment, with identical walking-meditation instructions	↓ Stress ↑ Mood ↑ Mindfulness ↑ Sleep quality (= in both interventions) = Nature connectedness
Owens et al., 2022 (UK) [29]	University students (*n* = 68)	20-min audio-guided mindfulness-compassion meditation with nature-based content outdoors over 2 weeks vs. indoor meditation without nature-based content vs. active control	↓ Rumination ↓ Depression ↑↑ Wellbeing (in nature > indoor/control)
**Quasi-Experimental/Pre–Post Studies**			
Lee et al., 2025 (Republic of Korea) [31]	Disaster-affected adults (*n* = 110)	2-day intensive forest therapy programme including forest mindfulness, sensory regulation and group activity	↓↓ Stress ↓ Anxiety ↓ Depression ↑ Vitality ↑ Mindfulness
Barrett et al., 2024 (US) [33]	Community and clinical samples (*n* = 64)	6–8-week Mindful EcoWellness programme integrating MBSR-based practices with sustainability education	↓ Anxiety ↓ Depression ↓ Fatigue ↑ Physical functioning Pro-environmental behaviours ↑ (exploratory)
Owens et al., 2025 (UK) [32]	Adolescents and young adults (*n* = 77)	13-min brief guided nature-based mindfulness meditation	↓ Rumination ↓ Stress (small age differences)
Kang et al., 2023 (South Korea) [30]	Vulnerable populations (*n* = 276)	15-week therapeutic gardening programme combining mindfulness, sensory awareness and nature engagement	↓ Depression+ ↓ Anxiety+ ↓ Stress+ Mindfulness+ Life satisfaction+
**Observational/Experimental (Non-RCT)**			
Lymeus et al., 2018 (Sweden) [34]	Stressed university students (*n* ≈ 160 across studies)	Restoration Skills Training (ReST): open-monitoring mindfulness in botanical garden vs. indoor focused-attention training	↑ Attention ↑↑ Relaxation ↓ Stress (ReST outdoor > CMT indoor)
Liang et al., 2025 (China) [35]	Adults (*n* = 60)	Landscape-based meditation in temple setting, comparing water, forest, rock scenes, and with/without creative reflection	↓ Heart rate ↑ Relaxation ↑ Mood (>in water landscapes) ↑ Cognitive-emotional engagement (>with integrated literary creation tasks)
**Qualitative Studies**			
Djernis et al., 2023 (Denmark) [37]	Stressed university students (*n* = 6)	5-day forest-based mindfulness retreat	↑ Self-regulation ↑ Mindfulness ↑ Emotional balance ↑ Connection ↑ Psycho-physical wellbeing
Schuling et al., 2018 (The Netherlands) [36]	Adults with recurrent depression (*n* = 15)	Multi-day silent mindful walking along river landscape	↓ Rumination ↑ Mindfulness ↑ Insight ↑ Wellbeing

Legend: ↑ improvement/increase; ↓ reduction; = no significant change; ↑↑/↓↓ comparatively stronger effect.

## Data Availability

No new data were created in this study.

## Appendix A

**Table A1:** Detailed study designs, interventions description, methodological characteristics and results of included nature-based mindfulness intervention studies. Studies are reported in the same order as Table 1. Abbreviations are defined at first use.

Author, Publication Year, Country	Study Design	Study Aims	Intervention—Detailed Description	Outcomes Measurements, Tools, Statistical Analysis	Results
**Randomised Controlled Trials**					
Choe, 2020, UK [26]	*Randomised experimental study with three parallel arms in* *different environments:* *- natural outdoor* *- built outdoor* *- indoor* *repeated measures at baseline, mid-intervention, post-intervention and 1-month follow-up.*	To test whether MBSR delivered in a natural environment enhances mental-health and wellbeing outcomes of university students and staff compared to built outdoor and indoor settings. To investigate whether the outcomes of MBSR are mediated by change in nature connectedness over time.	6-week brief MBSR programme (weekly 1-h sessions). Each session included standard mindfulness practices—guided seated meditation, mindful movement, walking meditation, and group reflection. The curriculum was identical across groups; only the delivery environment differed, allowing isolation of environmental effects.	**Outcomes:** - Mindfulness (FFMQ-SF); - rumination/reflection (RRQ); - depression, anxiety, stress (DASS-21); - nature connectedness. **Statistical Analyses:** SPSS 24 - χ^2^ tests and ANOVA to examine differences at baseline; - MANOVA across time points. —Follow-up analysis: one-way ANOVAs to compare environments, with post hoc comparisons (Tukey’s HSD). - Paired samples T-tests to investigate the impact of MBSR in each group. - mediation analysis (enhancement of MBSR outcomes by nature connectedness) examined by path analysis (Process macro for SPSS)	Participants in all three MBSR conditions (natural outdoor, built outdoor, indoor) showed significant improvements, with ↓ over time in stress, rumination, and ↑ in mindfulness-related measures. However, participants in the natural outdoor condition experienced greater ↓ in perceived stress and rumination, as well as more sustained effects at follow-up, compared with built outdoor and indoor settings. Only the natural environment group demonstrated a significant ↑ in nature connectedness, which mediated changes in reflective attitudes.
Djernis, 2021, Denmark [27]	*Three-arm pilot RCT comparing:* *- nature-based retreat* *- indoor retreat* *- wait-list control Assessments post-intervention and at 3-month follow-up.*	To compare effects on stressed university students of a residential mindfulness retreat delivered in nature versus indoors.	5-day residential MBSR-based retreat, including formal sitting meditation, yoga, silent walking, and an all-day intensive practice. The nature-based retreat was conducted entirely in a therapeutic garden, with all formal and informal practices outdoors; the indoor retreat followed the same structure inside a university facility. *All participants slept in tents to maintain the retreat format.*	**Outcomes:** - Perceived stress (PSS); - self-compassion (SCS); - mindfulness (FFMQ-SF, BCT); - connectedness to nature (CNS). **Statistical Analyses:** SPSS 25 - Mixed linear models for group × time effects	Both the nature-based and indoor residential mindfulness retreats resulted in significant ↓ in perceived stress and ↑ in mindfulness and self-compassion immediately post-intervention. At 3-month follow-up, improvements were largely maintained in both intervention groups. The nature-based retreat produced significantly greater improvements in connectedness to nature, whereas stress and mindfulness outcomes did not differ significantly between the two active interventions. *Author notes: The lack of additional benefits in the outdoor condition may be attributable to contextual factors (*e.g., *weather, noise, perceived safety) and the restorative design of indoor setting. In addition, the inward-focused attentional style central to standard MBSR may limit engagement with environmental features. This suggests that more outward-oriented/open-awareness practices could be better aligned with nature-based delivery.*
Ma, 2023, United Kingdom [28]	*Two-arm parallel RCT comparing:* *- mindful walking in nature group* *- mindful walking in an urban environment group. Participants were assessed at 4 time points: pre-study baseline, pre-intervention, post-intervention, and follow-up five days after the intervention.*	To examine effects of mindful walking on sleep, mood, mindfulness and nature connectedness in university students with sleep disturbances during the COVID-19 pandemic, and compare effects in natural vs. urban environments.	7-day mindful walking intervention, one 35-min walk per day, practiced individually, during daylight. All participants received identical written walking-meditation instructions (focus on breath, bodily sensations and present-moment awareness). Walking routes differed by condition (urban park vs. busy commercial street)—the two routes were matched for distance (30–35 min at moderate pace) and safety.	**Outcomes:** – Sleep quality (PSQI) – Mood (POMS-SF) – Physical activity (IPAQ) – Nature Relatedness (NR-6) – Mindfulness (MAAS, TMS) **Statistical Analyses:** – Descriptive statistics, Shapiro–Wilk tests for normality. – Independent t-tests to check baseline group equivalence. – Repeated-measures ANOVAs (per Group × Time) – Additional analyses of state mindfulness and qualitative content analysis of open-ended feedback (perceived positive/negative aspects of the intervention).	From pre- to post-intervention, participants in both the nature and urban walking groups showed significant improvements in sleep quality, mood, and mindfulness. No significant group × time interactions were observed, indicating comparable benefits across environments under the conditions of this trial. At short follow-up, sleep quality partially deteriorated in both groups after cessation of walking, while mood and mindfulness gains were largely maintained. Qualitative feedback highlighted enjoyment of walking outdoor in nature as a valued contextual feature of the intervention, perceived as supporting motivation and adherence rather than as a direct mental-health outcome. Participants also noted practical challenges, including time and energy demands and crowded or noisy walking routes.
Owens, 2022, UK [29]	*Three-arm RCT comparing:* *- nature-based guided meditation outdoors* *- indoor guided meditation* *- active control (audio tour of a university building). Assessments at baseline, immediately post-session and at 2-week follow-up.*	To assess effects of a brief (20-min) self-guided audio meditation with nature-based content outdoors on rumination, depressive symptoms and mental wellbeing in university students over a two-week period, compared to a non-nature-based indoor meditation and an active control condition.	20-min audio-guided meditation over two weeks. Content integrated mindfulness of breath, compassion-focused soothing rhythm breathing, sensory engagement with surroundings and gentle movement. Nature specific language was used for the nature-based guided meditation outdoors. In the indoor meditation condition nature-specific language was replaced with indoor equivalents. The active control condition consisted of 20-min audio-guided tour of a busy university building.	**Outcomes:** – Rumination (RRS, BSRI). – Depressive symptoms (PHQ-8) – Mental wellbeing (SWEMWBS). **Statistical Analyses:** – Repeated-measures ANOVA (RM-ANOVA) to examine Group × Time interactions for RRS, BSRI (T0–T1), PHQ-8 and SWEMWBS (T0–T2). – Linear regression (per meditation group) to test whether number of meditation attempts predicted T2 PHQ-8 and SWEMWBS scores, controlling for baseline. – Minimal clinically important differences (MCID); number needed to treat (NNT = 1/ARR) estimated	Immediately following the intervention, state rumination ↓ across all groups; however, only the nature-based meditation group exhibited a significant ↓ in trait rumination. Over the 2-week follow-up, both meditation groups showed ↓ in depressive symptoms, but larger effects and clinically meaningful change thresholds were observed only in the nature-based condition. Mental wellbeing improved significantly in the nature-based group but not in the indoor or control groups. Dose–response analysis showed that, in the nature group, a greater number of meditation practices over the two weeks was significantly associated with ↑ wellbeing.
**Quasi-Experimental/Pre–Post Studies**					
Lee et al., 2025, Republic of Korea [31]	*Pre–post single-group feasibility study assessing the effect of a forest therapy programme incorporating mindfulness practices on individuals affected by natural disasters.* *Assessments immediately before and after the intervention.*	To examine the **feasibility** and **psychological benefits** of a forest therapy programme incorporating mindfulness practices for individuals affected by natural disasters. -A further aim was to investigate whether improvements in mindfulness would **moderate** changes in psychological distress	2-day intensive forest therapy programme, including mindful tasting (food preparation and sensory focus), forest-based mindfulness practices (breath awareness, relaxation), aimed at reducing trauma-related alterations in arousal and reactivity, and group physical activities to foster connection with nature and others.	**Outcomes** -Depression (MHS:D) -Anxiety (MHS:A) -Vitality/daily life engagement (CORE—5 items: sleep, eating, physical activity, intimate relationships, learning new things) -Mindfulness (FFMQ-SF) -Stress response: Stress Response Inventory—Short Form (somatization, anger, depression) **Statistical Analyses:** -Multilevel analysis (pre vs. post) - Effect sizes (Cohen’s d) for pre–post differences. -Moderation analyses (Time × mindfulness change) predicting changes in anxiety, stress, and depression.	Significant pre–post improvements were observed across all domains, with ↓ in depression, anxiety, and stress response, with medium-to-large effect sizes. Participants also demonstrated significant ↑ in vitality and mindfulness. Moderation analyses showed that greater ↑ in mindfulness were associated with larger ↓ in distress, supporting mindfulness as a key change process in disaster-affected populations.
Barrett et al., 2024, USA [33]	*Pre–post pilot studies testing a mindfulness-based eco-wellness programme (“Mindful Eco-Wellness”), focusing on feasibility, acceptability, and preliminary trends in health and pro-environmental behaviours:* *-two community-based feasibility pilots* *-four group medical visit (GMV) pilots in a clinical setting.*	To explore feasibility and preliminary outcomes of an eco-wellness group programme, simultaneously targeting individual’s health (e.g., diet, physical activity, mental health) and environmental sustainability (e.g., carbon footprint via diet and transportation).	6–8-week Mindful EcoWellness programme, based on MBSR, with weekly 2-h group sessions and home practice. Combined mindfulness meditation, mindful movement and outdoor practices with sustainability education (food, transport, energy, consumption). Teaching videos (short lectures by environmental scientists) support each theme; all materials are made freely available online.	**Outcomes:** -Health and functioning: **PROMIS-29** (physical function, anxiety, depression, fatigue, sleep disturbance, social roles, pain, global pain). -Affective/relational measure: Feeling Loved scale -Pro-environmental behaviour (PEB) for energy use, active transport, diet, water use, purchasing, plastic reduction, civic engagement **Statistical Analyses:** -Primarily descriptive and feasibility-focused. - pre–post Student t-tests for PROMIS-29, Feeling Loved, and continuous PEB measures. *No control groups; small sample sizes; emphasis on trends and practicality rather than inferential efficacy.*	Overall, the programme was **feasible and well-accepted**: -High attendance and retention across community and clinical cohorts. -Participants reported strong motivation to improve diet, physical activity, stress, and environmental impact. Across community and clinical pilot cohorts, participants demonstrated significant pre–post improvements in multiple physical-mental health domains, including anxiety, depression, fatigue, and physical functioning. Descriptive trends suggested ↑ in pro-environmental behaviours (e.g., dietary choices, transport habits), although these outcomes were exploratory and not evaluated within controlled designs.
Owens, 2025, UK [32]	*Single-group repeated-measures design (within-subjects) to assess effect of a single 13-min nature-based guided meditation on rumination and stress in adolescents and young adults. Assessments immediately before and after.*	To test effects of a brief nature-based meditation. To determine whether the effects differ between age groups.	13-min guided nature-based meditation, adapted for adolescents and young adults. Practice included grounding, breath awareness, multisensory noticing of nature and imagery (e.g., “being like a tree”). Delivered outdoors in green spaces (parks or similar, with grass and trees).	**Outcomes:** -State rumination (BSRI) -State stress (VAS) **Statistical analysis:** -Two separate RM-ANOVAs with Time (T0 vs. T1) as within-subjects factor and Group (adolescent vs. adult) as between-subjects factor, testing main effect of Time (overall change) and Group × Time interaction (age differences). -Cohen’s d calculated as effect size	Significant immediate ↓ in state rumination and stress were observed following the brief nature-based meditation in both adolescents and young adults, with somewhat larger effect sizes in young adults. The study provides preliminary support for using short, age-adapted nature-based mindfulness practices to improve mental wellbeing in adolescents and suggests potential for school- and university-based mental health promotion.
Kang, 2023, South Korea [30]	*Longitudinal observational pre–post study to assess the effect of a 30-session nature-based therapy (NBT) gardening programme* *-Participants assessed twice: at baseline (pre-intervention) and after the end of the intervention.*	To examine psychological mechanisms linking gardening-based therapy and wellbeing. To test whether mindfulness at post-intervention mediates the relationships between baseline depression, anxiety and stress and later life satisfaction.	15-week therapeutic gardening programme comprising 30 group sessions (two per week, 2 h each) across institutional gardens. Activities included mindful planting, harvesting, sensory awareness of soil and plants, and nature-based leisure (e.g., tea rituals, flower arrangement, light yoga). Programme content followed a manual from the Korea National Arboretum—reviewed by clinical psychologists and horticultural-therapy experts.	**Outcomes:** – Depression (MHS:D) – Anxiety (MHS:A) – Perceived stress (Korean-PSS) – Mindfulness (Korean-MAAS) – Life satisfaction (Korean-SWLS). **Statistical Analysis:** - Descriptive analyses - Pearson’s correlations between variables at T1 and T2 with Holm–Bonferroni correction. - Structural equation modelling with mediation model	Participants showed significant ↓ in depression, anxiety and perceived stress from baseline to post-intervention, alongside ↑ in mindfulness and life satisfaction. Structural equation modelling indicated that post-intervention mindfulness mediated the relationship between baseline psychological distress and later life satisfaction. Results suggest that mindfulness is a key mechanism linking nature-based gardening interventions to ↓depression and anxiety and ↑life satisfaction in vulnerable populations.
**Observational/Experimental (Non-RCT)**					
Lymeus, 2018, Sweden [34]	*Two non-randomised trials comparing **Restoration Skills Training (ReST)** with a structurally matched **Conventional Mindfulness Training (CMT)*** *Repeated measures before and after classes in weeks 1, 3, and 5.*	To examine whether a nature-based, low-effort open-monitoring mindfulness training (Restoration Skills Training: ReST) supports short-term attentional restoration and stress reduction, leads to generalised attentional improvements over several weeks, and performs comparably to or better than conventional indoor focused-attention mindfulness training (CMT), particularly in participants with stress or concentration difficulties.	Restoration Skills Training (ReST): 5-week course with weekly 90-min sessions conducted in a tropical greenhouse/botanical garden with high restorative qualities. The programme emphasised low-effort open-monitoring and multisensory exploration of the environment through grounding practices, modality-specific sensory exercises, and open sensory awareness while sitting or moving outdoors. Daily home practice (~15 min) used ReST-style exercises. Conventional Mindfulness Training (CMT) was delivered over the same duration and session length in indoor classrooms without external views, emphasising focused-attention practices (e.g., mindfulness of breath, eyes closed), monitoring distraction and sustained attention.	**Study 1:** **Outcome:** -Attention (LDST) -Emotional state: Swedish—SCAS Assessments before and after classes on weeks 1, 3, 5. **Data analysis**: -RM-ANOVA; followed by ANCOVA for post-class scores controlling for pre-class scores. **Study 2:** **Outcome:** -Attention: LDST (as above); Trail Making Test (TMT) parts A and B (20-s trials): -TMT-A: information processing speed; -TMT-B: set-switching; -Emotional state: SCAS Assessments at pre-course, and before/after 20-min meditation sessions on weeks 1, 3, 5. **Data analysis**: - RM-ANOVA with Course × Week × Time; - ANCOVA for change scores	Across both studies, participants in the ReST programme demonstrated significant improvements in attentional performance and emotional relaxation over time. Short-term attentional gains following sessions were consistently greater in the outdoor ReST condition than in indoor focused-attention training. Longer-term attentional improvements were comparable between groups, while ReST showed more favourable patterns of restoration without increased cognitive effort. Authors propose restoration as a distinct pathway for attentional benefits in early meditation training and suggest ReST as an undemanding introduction to mindfulness, especially suitable for people with stress and concentration problems or as a way to enhance restoration during limited nature contacts.
Liang, 2025, China [35]	*Field experimental study conducted in a Chan Buddhist temple comprising two within-subject experiments.* *(1) compared psychophysiological responses to three meditation* *-* *related landscapes (water/lake, forest, rock).* *Pre- and post-viewing mood and perceived relaxation were assessed.* *(2) used a repeated* *-* *measures crossover design in the most restorative landscape (lake), comparing landscape viewing alone vs. viewing combined with poetry composition.*	1. To determine which type of landscape (water, forest, or rock) optimally promotes physiological and psychological relaxation during meditation-like observation 2. to assess whether integrating literary creation (five-line nature poetry) during water-landscape meditation further enhances relaxation and cognitive-emotional engagement.	Landscape-based meditation conducted in a Buddhist temple, comparing water, forest and rock landscapes. In a second experiment, participants composed short nature-poems during observation (only in water landscape, identified as optimal in Experiment 1), to enhance reflective engagement.	**Outcomes:** - Heart rate monitoring (bpm) - Self-determined observation duration (seconds) for each scene/condition - mood (POMS 2–Adult, Chinese version), pre- and post-viewing for each scene. -A rated questionnaire (8 items, 5-point Likert scale) assessed liking, desire to revisit, perceived relaxation, and attraction of colours, materials, plants/animals, details and overall scene; plus open-ended comments. **Statistical Analysis:** - Repeated-measures ANOVAs with Bonferroni post hoc tests compared scenes and time points for HR and POMS scores. - Correlations analysis tested associations between observation duration, HR changes, POMS differences and preference scores,—In Experiment 2, paired-samples and independent-samples t-tests (Cohen’s d) compared NP vs. WP and subgroups (deep reflection vs. non-deep reflection).	Water-based landscapes elicited the greatest ↓ in heart rate and negative mood states compared with forest and rock scenes. Longer observation duration was associated with greater physiological relaxation. In the second experiment, the addition of in situ poetry composition enhanced emotional engagement and was associated with more efficient physiological relaxation, particularly among participants demonstrating deeper reflective engagement. Overall, the study suggests that carefully designed water landscapes and integrated literary creation tasks can optimise both relaxation and cognitive–emotional engagement in nature-based meditation interventions.
**Qualitative Studies**					
Djernis, 2023, Denmark [37]	*Qualitative study using interpretative phenomenological analysis (IPA) nested within an RCT (Djernis, 2021* [27]).	To explore experiences of self-regulation in nature-based residential mindfulness retreat, exploring what was most helpful (mindfulness training, the natural environment, the group) and how they made sense of these experiences over time	5-day residential mindfulness retreat based on the standard 8-week MBSR curriculum. -All activities took place outdoors in a 3.5-acre forest therapy garden (Nacadia^®^) in an arboretum, designed specifically for nature-based interventions. -Participants stayed in individual tents on site -all formal practice, breaks and meals were outdoors. -Weather during the retreat was mostly sunny, 10–30 °C	**Qualitative outcomes:** - semi-structured, open-ended interviews post-retreat and at 3-month follow-up (focus on challenging real-life situations and concrete experiences of support for self-regulation, what was helpful in dealing with stress) **Analysis**: - Interpretative Phenomenological Analysis (IPA)	Four interrelated themes describing enhanced self-regulation emerged: (1) supportive conditions, including retreat setting and immersion in nature, fostered calm, presence and reduced decision load; (2) application of mindfulness attitudes (e.g., non-judgement, acceptance) supported self-acceptance and tolerance of difficult experiences; (3) strengthened connections to oneself, others and nature were described as crucial during emotional challenges; (4) improved physical and psychological balance (e.g., calm, focus, energy and insight). At 3-month follow-up, participants reported sustained benefits, including improved concentration, boundary setting and perceived resilience.
Schuling, 2018, The Netherlands [36]	*Qualitative study using thematic analysis of a multi-day nature-based mindful walking programme (“Het Pad”), drawing on semi-structured telephone interviews conducted 6–9 months post-retreat and triangulated with participant diaries and written evaluation forms.*	To explore which design characteristics and experience-based processes contribute to the positive effects of a multiday silent mindful walking retreat. To derive practical implications for designing effective mindful walking retreats as part of a mindfulness portfolio.	Multi-day silent mindful walking retreat along river landscapes, combining solo/group walking, silence and teacher guidance.	**Qualitative outcomes:** - Semi-structured interviews (20–60 min, recorded and transcribed verbatim) guided by a topic list exploring experiences during the walk, perceived helpful/hindering aspects, and perceived changes. -Daily diaries completed by participants **Analysis:** -Thematic analysis -Three researchers independently coded each transcript -Data triangulation with diaries and evaluation forms to validate themes and check for contradictions (none found)	Participants identified key features supporting psychological change: *Design factors*: flexible programme organisation, immersion in a varied river landscape supporting tranquillity and sensory awareness, multi-day walking fostering a sustained bodily rhythm, and the combination of silence with supportive guidance from mindfulness teachers. *Experiential processes:* ↑ awareness of thoughts and sensory perceptions, greater insight, ↓ rumination, letting go of habitual patterns, and improvements in wellbeing. Participants described being in nature, extended walking, silence, and mindfulness practices as mutually reinforcing, and viewed the programme as a valuable standalone or complementary alternative to conventional mindfulness courses.

Legend: ↑ improvement/increase; ↓ reduction.
